# Work hardening behavior of hot-rolled metastable Fe_50_Co_25_Ni_10_Al_5_Ti_5_Mo_5_ medium-entropy alloy: in situ neutron diffraction analysis

**DOI:** 10.1080/14686996.2022.2122868

**Published:** 2022-09-26

**Authors:** Hyeonseok Kwon, Stefanus Harjo, Takuro Kawasaki, Wu Gong, Sang Guk Jeong, Eun Seong Kim, Praveen Sathiyamoorthi, Hidemi Kato, Hyoung Seop Kim

**Affiliations:** a Department of Materials Science and Engineering, Pohang University of Science and Technology (POSTECH), Pohang, Republic of Korea; b J-PARC Center, Japan Atomic Energy Agency, Ibaraki, Japan; c Department of Metallurgical Engineering, Indian Institute of Technology (BHU), Varanasi, India; d Institute for Materials Research, Tohoku University, Sendai, Japan; e Center for Heterogenic Metal Additive Manufacturing, Pohang University of Science and Technology (POSTECH), Pohang, Republic of Korea; f Graduate Institute of Ferrous and Energy materials Technology (GIFT), Pohang University of Science and Technology (POSTECH), Pohang, Republic of Korea

**Keywords:** In situ neutron diffraction, medium-entropy alloy, work hardening, tensile strength, martensitic transformation, lattice strain, phase stress

## Abstract

Metastability engineering is a strategy to enhance the strength and ductility of alloys via deliberately lowering phase stability and prompting deformation-induced martensitic transformation. The advantages of the strategy are widely exploited by ferrous medium-entropy alloys (MEAs) that exhibit phase transformation from metastable face-centered cubic (FCC) to hexagonal close-packed (HCP) or body-centered cubic (BCC) martensite and a significant increase in work hardening. Fe_50_Co_25_Ni_10_Al_5_Ti_5_Mo_5_ (at%) MEA is an example of such materials, which shows ~1.5 GPa of tensile strength assisted by exceptional work hardening from the deformation-induced BCC martensitic transformation. In this work, the martensitic transformation and its effect on the mechanical response of the MEA were studied by in situ neutron diffraction under tensile loading. Strain-induced BCC martensite started forming rapidly from the beginning of plastic deformation, reaching a phase fraction of ~100% when deformed to ~10% of true strain. Lattice strain and phase stress evolution indicate that stress was dynamically partitioned onto the newly formed BCC martensite, which is responsible for the work hardening response and high flow stress of the MEA. This work shows how great a role FCC to BCC martensitic transformation can play in enhancing the mechanical properties of ferrous MEAs.

## GRAPHICAL ABSTRACT

**Figure:**
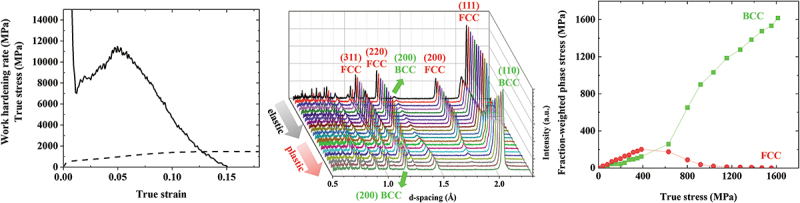

## 1. Introduction

High-entropy alloys (HEAs) are an emerging group of metallic materials that are capturing a great deal of attention for their unique design principle. The HEAs contain multiple principal elements that form a solid solution, which makes them differ from conventional alloys consisting of one principal element and other minor elements [1–3]. The compositional feature accompanies higher configurational entropy (ΔS_conf_) than conventional alloys, which enables classifying all alloys into three groups: HEAs, medium-entropy alloys (MEAs), and low-entropy alloys (LEAs). That is,

(1) $$\Delta S_{conf}\;\geq 1.5R\;for\;the\;HEAs,$$

(2) $$1.5R>\Delta S_{conf}\geq 1.0R\;for\;the\;MEAs,$$

(3) $$1.0R>\Delta S_{conf}\;for\;the\;LEAs,$$

where R is the gas constant, 8.315 J·K^−1^·mol^−1^ [1].

The unique design concept is not the only merit of the HEAs. The HEAs possess attractive mechanical properties largely attributed to the massive solid solution strengthening originating from the multicomponent feature, as they are complex concentrated solid solutions formed by atoms with various atomic radii. In addition, other strengthening methods such as precipitation [4,5], grain refinement [6], gradient structuring [7,8], mechanical twinning [9,10], or mechanical phase transformation [11] can come into play for further enhancement in mechanical properties. Among them, the mechanical twinning and phase transformation during deformation effectively help improve work hardening ability and have been utilized by controlling the stacking fault energy of the alloys, which determines phase stability and deformation mechanism.

Ferrous MEAs with increased Fe contents (≥50 at%), which are being widely investigated recently, represent the materials that exploit the advantages of mechanical phase transformation. Many ferrous MEAs developed so far show metastable face-centered cubic (FCC) matrix, which easily transforms into hexagonal close-packed (HCP) or body-centered cubic (BCC) structures under deformation [12–16]. This behavior has been the key to synergetic improvement of strength and ductility in these materials and harnessed under the name of ‘metastability engineering’. Fe_50_Co_25_Ni_10_Al_5_Ti_5_Mo_5_ (at%) MEA previously reported by the present authors is an example of the metastable ferrous MEAs [17,18]. After hot rolling at 1150℃ followed by air cooling, the MEA consists of metastable FCC, thermally induced BCC martensite, and Mo-rich µ-phase precipitates. The average size of the prior equiaxed FCC grains was 60 ~ 70 µm, and the thermally induced BCC martensite with lath structure formed during the cooling had an average block size of ~4.4 µm. Both FCC and BCC phases contained a high density of dislocations [17]. Upon tensile deformation, the retained FCC phase goes through mechanical phase transformation into BCC martensite. The martensitic transformation involves an abrupt increase in work hardening rate and leads to ultimate tensile strength (UTS) of ~1.5 GPa and uniform elongation of ~15% [17]. It is not common to achieve such a work hardening ability and ultimate tensile strength in an as-rolled material, but a quantitative explanation of the deformation-induced martensitic deformation and its effect on the mechanical behavior was missing in our previous work.

In the present study, we employed in situ neutron diffraction tensile testing to understand the exceptional mechanical response of the Fe_50_Co_25_Ni_10_Al_5_Ti_5_Mo_5_ ferrous MEA. The development of FCC and BCC phases under tensile deformation at room temperature was investigated in real-time, and dynamic stress partitioning between the phases and its contribution to the work hardening behavior was quantitatively analyzed in terms of lattice strain and phase stress evolution.

## 2. Experimental procedure

A 7 × 35 × 60 mm^3^ ingot of the Fe_50_Co_25_Ni_10_Al_5_Ti_5_Mo_5_ MEA was cast by vacuum induction melting of alloying elements with purity higher than 99.9%. The ingot was homogenized at 1150℃ for 6 hours. The ingot was subsequently hot-rolled at the same temperature, with a thickness reduction ratio of ~79% (from 7 mm to 1.5 mm). The hot-rolled plate was air-cooled to room temperature. Tensile properties of the hot-rolled alloy were tested using tensile specimens with a gauge geometry of 6.4 × 2.5 × 1.5 mm^3^ fabricated along the rolling direction of the plate at a constant strain rate of 1 × 10^−3^ s^−1^ (Instron 1361). The digital image correlation (DIC) method was employed to measure tensile strain (ARAMIS M12). The tensile test was repeated at least three times to ensure reproducibility. Microstructures of the specimens were characterized by a field-emission scanning electron microscope (FE-SEM, Philips FEG XL30S) equipped with an electron backscatter diffraction (EBSD) detector. For the microstructural analysis, the samples were mechanically polished with SiC 600, 800, and 1200 grit papers and electropolished in a solution of 92% CH_3_COOH and 8% HClO_4_ to remove residual stress and martensite possibly induced by the mechanical polishing.

The in situ neutron diffraction experiment during tension was conducted with a high-resolution time-of-flight neutron diffractometer for engineering materials science at BL19 ‘TAKUMI’ of Materials and Life Science Experimental Facility of Japan Proton Accelerator Research Complex. The details about the facility can be found in Ref [19]. The tensile specimen for the experiment was cut along the rolling direction of the hot-rolled plate into a gauge geometry of 25 × 4 × 1.5 mm^3^. The tensile specimen was loaded in a step-load controlling mode during the elastic deformation and a step-displacement controlling mode during the plastic deformation. The load rate in the elastic regime was 60 N/s, and the strain rate in the plastic regime was 1 × 10^−3^ s^−1^. The loading was paused for 10 minutes at each step to collect the diffraction data. After reaching the end of uniform elongation, the specimen was unloaded to 0 N for measurement of residual lattice strain. The tensile direction was at 45° with the incident beam, and two detector banks with collimators at +90° and −90° with the incident beam (axial and transverse directions of the specimen, respectively) acquired the diffraction data. Strain gauges glued on the surface in a way they were separated from each other for 25 mm were used to measure tensile strain. The axial diffraction data were utilized for lattice strain and phase stress analysis of this study.

## 3. Results and discussion

### 3.1. Mechanical behavior

**Figure 1.**
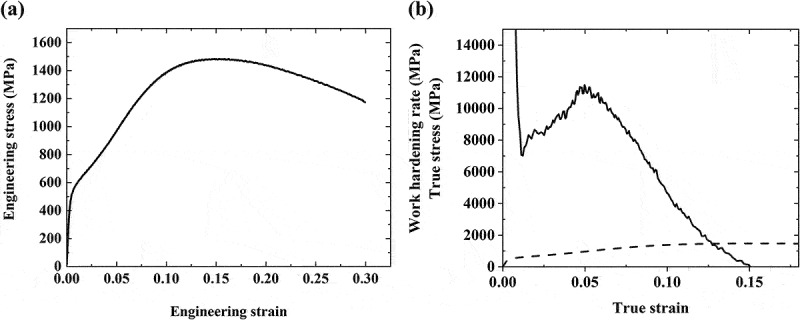
(a) Engineering stress–strain plot. (b) Work hardening rate and true stress–strain curve of the Fe_50_Co_25_Ni_10_Al_5_Ti_5_Mo_5_ MEA.

The tensile properties of the hot-rolled Fe_50_Co_25_Ni_10_Al_5_Ti_5_Mo_5_ MEA are shown in Figure 1(a). The MEA exhibits 528 ± 38 MPa of yield strength (YS), 1461 ± 17 MPa of UTS, and 13.0 ± 0.7% of uniform elongation with 26.0 ± 4.1% of total elongation. The high post-necking elongation due to the short gauge length of the sample partly accounts for the high total elongation. Strength increment from the onset of the plastic deformation until the UTS is close to ~1 GPa. Work hardening rate (WHR) and true stress plotted against true strain in Figure 1(b) depict the work hardening ability of the MEA, with the WHR starting to increase immediately after the yield point and exceeding ~11 GPa at its highest.

### 3.2. Phase evolution

**Figure 2.**
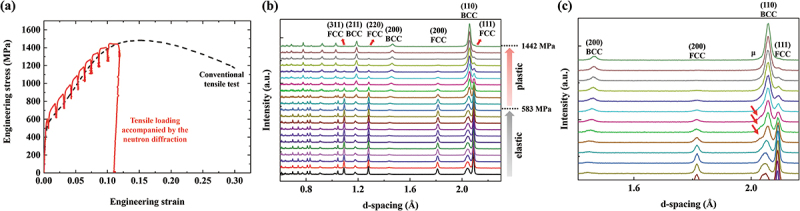
(a) Engineering stress–strain plot obtained from the tensile loading accompanied by neutron diffraction and conventional tensile test. (b) Neutron diffraction patterns taken under the tensile loading. (c) An enlarged version of the plastic deformation regime of (b) that highlights minor µ phase peaks and FCC/BCC peak position shifts.

The engineering stress–strain curve obtained from the in situ neutron diffraction tensile loading in Figure 2(a) shows a good agreement with the conventional tensile test. The neutron diffraction patterns are displayed in Figure 2(b). The patterns were collected up to the end of uniform elongation, which approximately corresponded to ~1443 MPa of engineering stress. There are retained FCC and BCC martensite co-existing from the initial state before the deformation. The lattice parameters of the FCC and BCC phases before deformation are ~3.616 and ~2.889 Å, respectively. As deformation proceeds, the BCC phase peaks gradually increase in intensity, while those of the FCC phase decrease. The FCC peaks dramatically diminish after the onset of the plastic deformation and nearly vanish at the end. The enlarged version of the patterns in Figure 2(c) shows the peak of the Mo-rich µ phase located close to the (110) BCC peak. The peak stands for (10$\bar{1}$1) hexagonal close-packed (HCP) structure [17]. At lower strains, the µ phase peak is not clearly discernible as it overlaps with the nearby (110) BCC peak. In the diffraction patterns taken at higher strains, the (110) BCC peak grows in intensity due to the DIMT, and there is a certain range where the µ phase peak is more clearly distinguished, which is marked with the red arrows. However, in the later stage of deformation, the (110) BCC peak broadens, making it difficult to observe the µ phase peak. On the other hand, the FCC and BCC peaks clearly shift to higher d-spacing with the deformation, which is related to the lattice strain evolution that will be covered in detail later.

**Figure 3.**
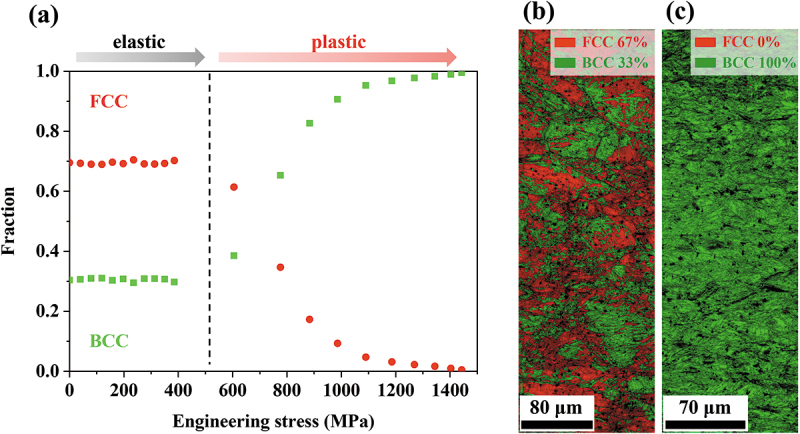
(a) FCC and BCC phase fraction evolution plotted against engineering stress. EBSD phase analysis of the Fe_50_Co_25_Ni_10_Al_5_Ti_5_Mo_5_ MEA (b) before tensile loading and (c) deformed to ε_loc_ = ~13%.

The fractions of the FCC and BCC phases can be evaluated by an empirical equation that has been used for maraging stainless steel [20]:

(4) $$f_{BCC}=\frac{A_{\left(110\right)BCC}}{A_{\left(110\right)BCC}+1.4\cdot A_{\left(111\right)FCC}},$$

where $f_{BCC}$ is phase fraction of BCC, $A_{\left(110\right)BCC}$ and $A_{\left(111\right)FCC}$ are peak areas of the (110) BCC and (111) FCC, respectively. The measured phase fractions are plotted against applied engineering stress in Figure 3(a). In the elastic deformation regime, the phase fractions hardly change. However, the sharp decrease in FCC and increase in BCC occur at the onset of the plastic deformation. This indicates that a strain-induced mechanism governs the martensitic transformation in the present MEA as opposed to stress-induced martensitic transformation, where the martensite starts forming before the yield point [12,21,22]. The EBSD phase maps enable direct observation of the phase evolution. The map taken at the initial state (Figure 3(b)) shows a microstructure comprising ~67% FCC and ~33% BCC, while that taken after tensile deformation to a local strain of ~13% (Figure 3(c)) exhibits nearly full BCC martensite. The tendency is in good agreement with the information from the diffraction data.

The rapid martensitic transformation can be attributed to the microstructural features of the present hot-rolled MEA. In our previous report, elemental analysis using transmission electron microscopy (TEM) revealed that the composition of the FCC/BCC matrix after the μ phase precipitation is approximately Fe_52.17_Co_26.59_Ni_9.21_Al_2.45_Ti_4.52_Mo_5.06_ (at%) [17]. Gibbs free energy difference between the FCC and BCC (ΔG_FCC→BCC_, defined as G_BCC_-G_FCC_), commonly used as an indicator of phase stability of alloys that governs martensitic transformation along with stacking fault energy, was calculated as −3.5 kJ/mol based on the matrix composition. The negative value shows that the BCC phase is supposed to be more stable than the FCC at room temperature, and the present FCC is thus metastable. The value is even lower than that estimated based on the bulk composition (−2.8 kJ/mol), which means the precipitation and corresponding deviation in the matrix composition have further reduced the FCC phase stability and facilitated martensitic transformation into BCC [14,23]. High dislocation density produced by hot rolling also plays a major role here [24–26]. According to the lattice parameters of the retained FCC and thermally induced BCC martensite after rolling, the volume of the BCC martensite is ~1.02 times bigger than that of the FCC phase [27]. The volume change needs to be accommodated by generating geometrically necessary dislocations (GND) on the FCC sides of the phase interfaces. The generated GNDs create back stress that stimulates the martensitic transformation [24,28]. High strain field and associated energy owing to the high dislocation density are also reported to increase the driving force for the martensitic transformation [24,29]. The dislocation interaction and martensitic transformation need to be further investigated by direct observation techniques on a nanometer scale such as TEM to gain a more thorough insight into the relationship between the microstructural features and mechanism.

### 3.3. Lattice strain evolution

**Figure 4.**
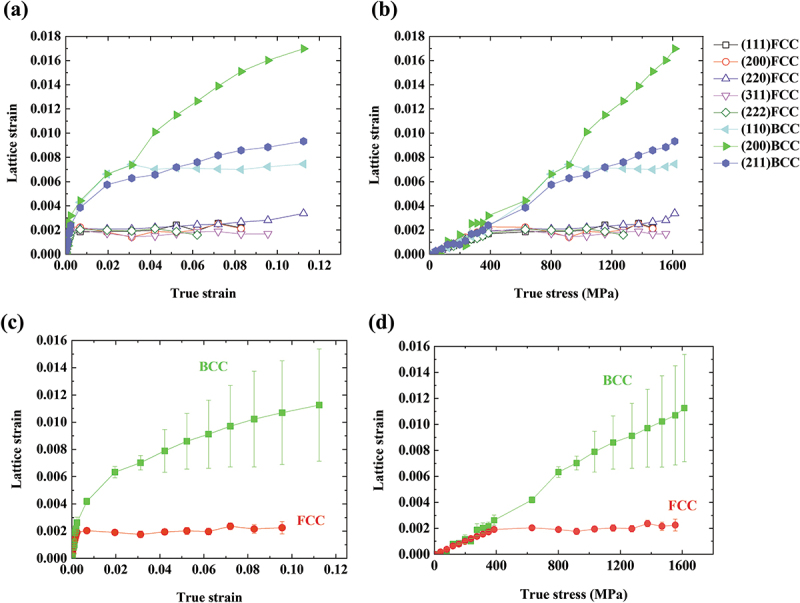
Lattice strain evolution depending on crystal orientations plotted against (a) true strain and (b) true stress. Averaged phase lattice strain evolution plotted against (c) true strain and (d) true stress.

From the loading-induced changes in lattice spacings and peak shifts from the original positions, the lattice strains of an (hkl) plane can be extracted as follows:

(5) $$\varepsilon_{hkl}=\frac{d_{hkl}-d_{hkl}^{0}}{d_{hkl}^{0}},$$

where $\varepsilon_{hkl}$ is the lattice strain, $d_{hkl}$ is the lattice spacing, and $d_{hkl}^{0}$ is the initial lattice spacing before tensile loading. The lattice spacings were computed by the pseudo-Voigt fitting of each peak. Figure 4 illustrates lattice strains of the FCC and BCC phases in the axial direction plotted against true strain (Figure 4(a)) and true stress (Figure 4(b)). The evolution of lattice strain varies significantly by each phase and crystal orientation. Since the beginning of the plastic deformation, the lattice strains of the BCC phase have increased drastically compared to those of FCC. The crystal orientations determine the increasing trends as well. In the BCC phase, the lattice strains of {200} planes increase faster than {211} and {110} planes and reach the highest values, while the {110} planes have the lowest lattice strains. This means that the {200} grains act as hard grains that take a larger load and the {110} grains are soft grains, which aligns with typical behaviors of BCC metals [27,30,31]. On the other hand, FCC planes do not show a noticeable variation depending on the orientations, with lattice strains of all grain families staying in low values. This is attributed to the accelerated martensitic transformation into BCC martensite, which is harder than the parent FCC and takes up a far bigger share of the load. The phase lattice strains of the FCC and BCC can be calculated by averaging the {hkl} lattice strains of each phase and are plotted against true strain (Figure 4(c)) and true stress (Figure 4(d)). One can affirm the development of high phase lattice strain of BCC that greatly exceeds FCC. The error gaps are also much larger in the BCC phase than in the FCC, which reflects the substantial difference in lattice strain evolution by the crystal orientations in the BCC. Depending on the crystal orientations, −0.0009, 0.0056, and 0.0012 of lattice strains were measured in {110}, {200}, and {211} BCC grain families, respectively, after unloading to 0 N. The average value of ~0.0020 indicates that tensile residual strain is retained in the specimen. The current results provide an insight that a salient stress−strain partitioning between the FCC and BCC phases dynamically evolves with martensitic transformation and greatly affects plastic deformation. Evaluation of phase stresses must be followed to quantify each phase’s contribution to the work hardening and flow stress.

### 3.4. Phase stress evolution

**Figure 5.**
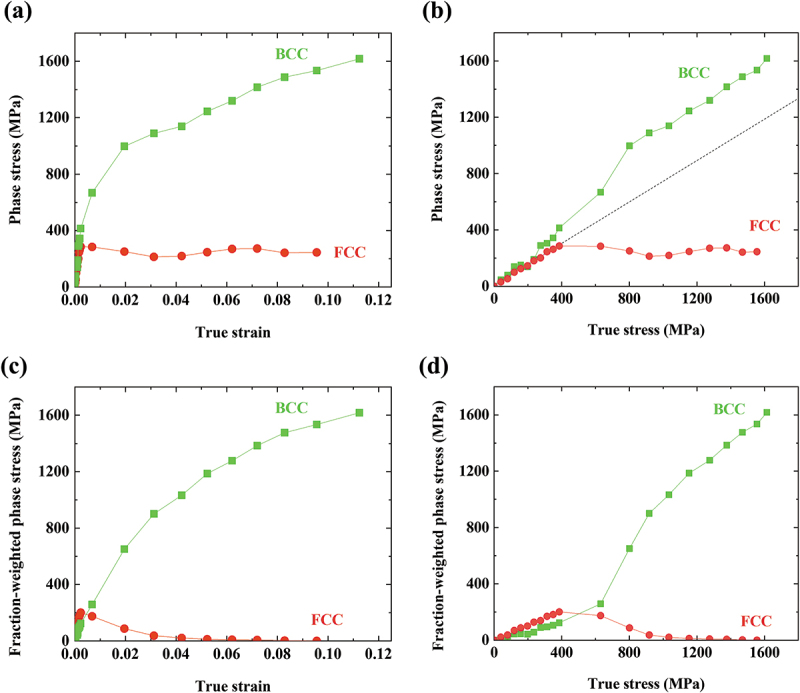
Phase stress evolution plotted against (a) true strain and (b) true stress. Fraction-weighted phase stress calculated by multiplying the phase stress by phase fraction plotted against (c) true strain and (d) true stress.

**Figure 6.**
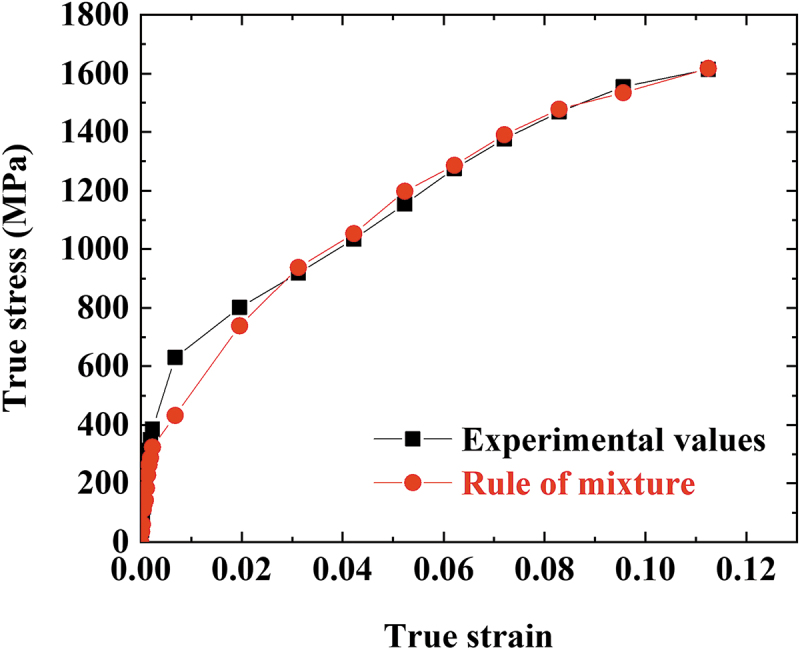
Comparison between experimentally measured stress and calculated stress by the rule of mixtures of phase stresses.

The phase stresses of the FCC and BCC phases can be acquired by Hooke’s law, as follows:

(6) $$\sigma_{FCC}=E_{FCC}\varepsilon_{FCC}^{\left(311\right)}\;\;for\;\;FCC,$$

(7) $$\sigma_{BCC}=E_{BCC}\varepsilon_{BCC}^{\left(211\right)}\;\;for\;\;BCC,$$

where $\sigma_{FCC}$ and $\sigma_{BCC}$ are the phase stresses of the FCC and BCC, respectively, and $E_{FCC}$ and $E_{BCC}$ are Young’s moduli of the FCC and BCC, respectively. $\varepsilon_{FCC}^{\left(311\right)}$ is the lattice strain of {311} planes of the FCC phase, and $\varepsilon_{BCC}^{\left(211\right)}$ is that of {211} planes of the BCC phase. It is well established that the $\varepsilon_{FCC}^{\left(311\right)}$ and $\varepsilon_{BCC}^{\left(211\right)}$ values can represent the overall behaviors of the FCC and BCC polycrystalline materials, relatively unaffected by intergranular strain [27,30,32,33]. To obtain the phase stresses from Equations (6) and (7), the calculation of $E_{FCC}$ and $E_{BCC}$ must be preceded. The initial microstructure of the present alloy consists of a dual-phase, making it difficult to determine Young’s modulus of each phase. So, $E_{BCC}$ must be obtained first from the data at the unloading stage, where the matrix has transformed into full BCC. After the flow stress and lattice strain of BCC reach maximum values of 1614 MPa and ~0.0113, respectively, the sample is unloaded to 0 MPa, and residual lattice strain of ~0.0020 remains in the BCC phase. The $E_{BCC}$ calculated from the decrement of stress divided by that of lattice strain, (1614–0)/(0.0113–0.0020) MPa, is ~173.4 GPa. And $E_{FCC}$ can be accordingly calculated to be ~144.9 GPa from the rule of mixture in the elastic deformation regime. The relatively low Young’s moduli compared to those of austenite and martensite phases of TRIP steel (~200 GPa and ~210 GPa, respectively) [27] can be attributed to the high dislocation density in the present alloy, as dislocation lines between pinning points such as point defects or other dislocations can bow out under stress and give extra elastic deformation [34]. The phase stresses determined using the Young’s moduli are plotted against true strain and true stress in Figure 5(a,b). The dynamic stress partitioning onto the BCC phase is clearly observed, as the $\sigma_{BCC}$ is way higher than the $\sigma_{FCC}$ over the entire deformation. The deviation of $\sigma_{FCC}$ to lower values than the elastic limit (Figure 5(b)) indicates the FCC phase is under compressive stress due to the formation of BCC with higher lattice volume. The contrast in the phase stress is more prominent when phase fractions are considered. The $\sigma_{BCC}$ and $\sigma_{FCC}$ multiplied by phase fractions are plotted in Figure 5(c,d) and highlight the gaps between the phase stresses that grow further as the deformation proceeds. The estimation of phase stresses can be verified by comparison with the experimentally measured bulk stress. The overall stress is calculated from the phase stresses by the rule of mixtures (ROM):

(8) $$\sigma_{ROM}=\sigma_{FCC}f_{FCC}+\sigma_{BCC}f_{BCC},$$

where $\sigma_{ROM}$ is the overall stress. The $\sigma_{ROM}$ plotted with experimental stress values in Figure 6 shows excellent agreement between the experiment and calculation, confirming the validity of a series of computations implemented in the present study.

The phase stress evolution demonstrates that the dynamically formed BCC phase takes far higher stress than the FCC and is responsible for most of the work hardening and flow stress. It is noteworthy that even after the microstructure reaches nearly 100% BCC, the work hardening rate and bulk flow stress still increase. This proves that the BCC martensite of the present MEA is not totally brittle and can accommodate a large share of plastic strain and deformation-induced hardening. This may be due to BCC martensite in C-free ferrous MEAs, which carries no tetragonality and does not lead to ductile-to-brittle transition, unlike the martensite in C-containing steels [12,35]. Figures 4(b) and 5(b) enable the observation of a decrease in the slopes of the lattice strain and phase stress of the BCC phase when the true stress and true strain exceeds 800 MPa and 0.05 true strain, respectively. This is in good agreement with the maximum value of the work hardening rate observed when the true stress and true strain exceed 800 MPa and 0.05 true strain, respectively, in Figure 1. These results indicate that the BCC martensite starts undergoing plastic deformation when the true stress is over 800 MPa, which must involve work hardening. Therefore, the superimposition of the stress partitioning effect from the elastic deformation of the BCC martensite and the large work hardening rate of the martensite results in the extraordinary work hardening response of the present hot-rolled MEA.

## 4. Conclusion

To summarize the present study, the effect of deformation-induced martensitic transformation on the mechanical response of hot-rolled Fe_50_Co_25_Ni_10_Al_5_Ti_5_Mo_5_ MEA was quantitatively investigated with the aid of in situ neutron diffraction measurement under tensile loading. The MEA consists of a dual phase matrix of retained FCC and thermally induced BCC martensite and shows outstanding work hardening ability and high UTS of ~1.5 GPa with uniform elongation of ~15%, which is unusual considering that it is in as-rolled condition with high dislocation density. The conclusions drawn from the in situ neutron diffraction study are as follows:

1. The neutron diffraction patterns showed that the retained FCC in the microstructure undergoes accelerated phase transformation into BCC martensite from the onset of plastic deformation and almost fully BCC structure is attained after being deformed to a true strain of ~0.1.
2. The newly formed BCC martensite exhibits a much higher lattice strain than the retained FCC, revealing that dynamic load partitioning occurs considerably between the two phases.
3. Phase stress evolution confirms that stress is distinctly partitioned onto the BCC martensite and accounts for most of the extreme work hardening behavior and high flow stress of the present MEA. Flow stress calculated by the rule of mixtures of the FCC and BCC phases showed good accordance with the experimentally measured flow stress.
